# Cardiometabolic risk indicators in individuals with bipolar disorders: a replication study

**DOI:** 10.1186/s13098-023-01044-7

**Published:** 2023-04-03

**Authors:** Hemen Najar, Alina Karanti, Erik Pålsson, Mikael Landén

**Affiliations:** 1 Institute of Neuroscience and Physiology, Section of Psychiatry and Neurochemistry, Sahlgrenska Academy, University of Gothenburg, Blå stråket 15, Gothenburg, 413 45 Sweden; 2 Department of Medical Epidemiology and Biostatistics, Karolinska Institutet, Stockholm, Sweden

**Keywords:** Bipolar disorder, Cardiovascular disease, Body mass index, Waist hip ratio, Blood pressure

## Abstract

**Objectives:**

We recently conducted the first longitudinal study comparing cardiometabolic risk indicators (CMRIs) between a cohort of individuals with bipolar disorders (BDs) and controls from the general population. Here, we sought to validate the findings in that study using an independent case-control sample.

**Methods:**

We used data from the St. Göran project’s Gothenburg cohort. The BDs group and the control group were examined at baseline and after a median of eight and seven years, respectively. Data collection occurred between March 2009 and June 2022. We used multiple imputation to handle missing data and linear mixed effects model to examine the annual change in CMRIs over the study period.

**Results:**

The baseline cohort included 407 individuals with BDs (mean age 40 years, 63% women) and 56 controls (mean age 43 years, 54% women). Of those, 63 persons with BDs and 42 controls participated at follow-up. At baseline, individuals with BDs had significantly higher mean values of body mass index (β = 0.14, p = 0.003) than controls. Over the study period, the difference in average annual change between the patient and the control group indicated an increase in patients relative to controls in waist-to-hip ratio (0.004 unit/year, p = 0.01), diastolic (0.6 mm Hg/year, p = 0.048), and systolic (0.8 mm Hg/year, p = 0.02) blood pressure.

**Conclusions:**

This study replicated the main findings from our previous study and showed that central obesity and measures of blood pressure worsened over a relatively short time in individuals with BDs relative to controls. It is vital for clinicians to monitor CMRIs in persons with BDs and to be proactive in preventing cardiometabolic diseases in this high-risk group.

**Supplementary Information:**

The online version contains supplementary material available at 10.1186/s13098-023-01044-7.

## Introduction

Individuals with bipolar disorders (BDs) are at increased risk for cardiometabolic diseases —like cardiovascular diseases (CVDs), hyperlipidemia, and type II diabetes mellitus— compared with the general population [1, 2]. This risk is to a large extent attributable to disturbances in lipid and glucose metabolism, increased prevalence of total and central obesity, and high blood pressure [1, 2]. It is well documented that these cardiometabolic disturbances can lead to reduced quality of life through impaired physical health [3], but also through negative effects on mental health and cognitive function [3–5], as well as through reduced social functioning [6]. Further, factors such as increased risk of disability pension, early retirement [7, 8], and sick leave [9] have a negative impact on personal finances. In fact, the risk for and duration of sick leave are increased even in those without CVD symptoms but with moderate to high CVD risk, as defined by the cluster of different cardiometabolic disturbances [9].

Studies that have shown poor cardiometabolic status in individuals with BDs have done so by analyzing the frequency with which they meet criteria for dyslipidemia [10], type II diabetes mellitus [11], hypertension [12], and total [13] and central obesity [14] compared with individuals in the general population. Fewer studies have analyzed differences in continuous cardiometabolic risk indicators (CMRIs) [15–17] like body mass index (BMI), waist circumference, systolic blood pressure (SBP), diastolic blood pressure (DBP), plasma triacylglycerol (TAG), total cholesterol (TChol), high-density lipoprotein-cholesterol (HDL-C), low-density lipoprotein-cholesterol (LDL-C), and plasma glucose. Such studies are, however, important because even small sub-threshold changes in individual CMRIs increase CVD risk [18–25]. And when combined, multiple CMRIs synergistically increase risk [26, 27].

We recently examined CMRIs in a Swedish cohort of individuals with BDs and general-population controls in the St. Göran Bipolar study [28]. We showed that individuals with BDs displayed higher levels of several CMRIs: waist-to-hip ratio (WHR), BMI, TAG, TAG/HDL-C ratio, TChol/HDL-C ratio, and non-HDL-C. Further, using data from a follow-up visit after a median of six to seven years, we found that WHR, SBP, and DBP worsened more in the BDs group than in the control group.

As our previous study was the first to examine central obesity, blood pressure, and atherogenic lipid profile over time in individuals with BDs, it needs to be replicated. In addition, the control group’s baseline blood pressure was unexpectedly higher than that of the patient group, making it difficult to interpret time-dependent blood pressure changes. We therefore aimed to replicate the results of our first study by examining the same CMRIs in an independent cohort using a nearly identical study design.

## Methods

The St. Göran Bipolar study is being conducted at two centers in Sweden. Our first study only used data from the Stockholm cohort because follow-up assessments were not available at the time in the second, Gothenburg, cohort. With follow-up data now available also in the Gothenburg cohort, we used these to replicate results from the Stockholm cohort. Study patients have been enrolled at the bipolar outpatient clinic in Gothenburg since March 2009.

A Swedish version of the Affective Disorder Evaluation (ADE) was used to diagnose all the patients. The ADE is a semi-structured diagnostic interview that was created for the Systematic Treatment Enhancement Program of Bipolar Disorder (STEP-BD) [29]. The ADE contains the affective module from SCID-I (Structured Clinical Interview for DSM-IV Axis I Disorders). Following a social anamnesis, the ADE collects information on lifetime affective episodes, suicide attempts, somatic illnesses, tobacco use, alcohol and drug use. Overall psychosocial functioning was evaluated by the symptom and function dimensions of the Global Assessment of Functioning (GAF) measures [30]. The Mini International Neuropsychiatric Interview (M.I.N.I.) was used to screen for psychiatric diagnoses other than BDs [31].

The St. Göran Bipolar study enrolls patients who are at least 18 years old, able to complete the standard clinical assessment, capable of providing informed consent, and who meet the DSM-IV (diagnostic and statistical manual of mental disorders- fourth edition) criteria for any bipolar spectrum disorder, i.e., bipolar I disorder, bipolar II disorder, bipolar disorder non-otherwise specified (NOS), cyclothymia disorder, or schizoaffective syndrome bipolar type. Individuals with BDs are not excluded upon having somatic and psychiatric comorbidities. In this study, as in the Stockholm study, we excluded individuals with schizoaffective disorder, bipolar type from the analysis [28].

Statistics Sweden randomly sampled and contacted population-based control individuals who were age- and sex-matched to patients that had been included until November 2012. No matching controls were sampled for patients included after November 2012. A research nurse screened the controls for the exclusion criteria: any current psychiatric disorder or any current use of psychotropic drugs, substance or alcohol abuse, neurological diseases (excluding mild migraine), untreated endocrine disorders, pregnancy, and schizophrenia or BD in first degree relatives. A psychiatrist interviewed the controls to screen for psychiatric disorders using M.I.N.I [31]. Alcohol use disorder and drug use disorder were assessed using AUDIT (the Alcohol Use Disorders Identification Test) [32] and DUDIT (the Drug Use Disorders Identification Test) [33], respectively.

Blood samples were obtained in the morning following a minimum eight-hour overnight fast. After blood sampling, the participants were served a light breakfast (a sandwich and a cup of coffee) followed by a physical examination. A research nurse measured blood pressure in the right arm using a manual sphygmomanometer (Henry Eriksson AB, cuff size 9 × 28 cm or 14 × 40 cm) in sitting or supine position after 15 min of rest. Weight was measured with light clothing and without shoes. Height was self-reported. Waist circumference was measured midway between the lower rib and the anterior superior iliac spine at the umbilical level. Hip circumference was measured around the widest portion of the gluteal region and hip. Weight, height, waist circumference, hip circumference, and blood pressure were measured to the nearest whole kg, cm, or mm Hg according to clinical praxis. All current use of medications was recorded.

All plasma lipid analyses were performed at the Clinical Chemistry Laboratory at the Sahlgrenska University Hospital, Mölndal, Sweden, on an Abbot Alinity ci-series photometric system. At baseline, the blood sampling and physical examination took place on separate occasions, separated by up to three months for all, except for 18 patients where the interval was up to six months. At follow-up, blood sampling and physical examination were performed on the same day, except for four controls with an interval of up to ten days.

The patient group had a follow-up visit after a median of eight years (ranging from six to twelve years). For the control group, the follow-up visit took place after a median of seven years (ranging from six to eight years). Blood sampling and physical examination were performed using the same protocol as for the baseline visit. Baseline observations were conducted March 2009 – June 2022 and follow-up observations were conducted April 2017 – April 2022.

To facilitate comparison with our previous study [28], we included the same CMRIs: WHR, BMI, SBP, DBP, TAG, TAG/HDL-C ratio, TChol/HDL-C ratio, and non–HDL-C.

The study was approved by the regional ethical review board in Stockholm, Sweden (registration code: 2008/1931.32). All participants consented orally and in writing after being presented with detailed information about the study.

### Statistical analysis

We used independent sample t-tests and linear regression models to examine group differences for continuous variables and chi-squared tests for categorical variables. Spearman’s correlation coefficient was used to examine correlation between different variables.

#### Imputation of missing data

At baseline, 1.8% in the control group and 45.7% in the BDs group had any missing baseline CMRI data. At follow-up, the percentages were 11.9% and 3.2% in the control and BDs group, respectively. We compared between individuals with complete data and those with missing data and plotted the frequency of missing data points against date of observation for each CMRI. More data were missing in the beginning of baseline recruitment and at the end of follow-up observation. Further, some observed variables were associated with missing data. For example, treatment with lithium and lamotrigine and having BD type 1 were more common in patients with complete data, while having cyclothymia or BD NOS, and lower SBP and DBP were more common in patients with missing data. Since the probability of having missing data partly depended on other observed data, but not on the values that were missing, we assumed a missing at random (MAR) mechanism [34]. Multiple imputation was chosen to handle missing data based on this assumption and the fact that more than 10% of the participants at each group lacked complete data [34].

We performed one set of imputations for baseline data and one for follow-up data. To enhance the results of the multiple imputation, we used an inclusive strategy [35] adding the following auxiliary variables [34, 35]: sex, age, case-control status, number of cigarettes per day, more/less than 12 years of education, working more/less than 50% time, GAF-scores, examination date, weight, height, hip circumference, waist circumference, SBP, DBP, TChol, TAG, HDL-C, LDL-C, creatinine, somatic illness (including hypertension, angina pectoris, myocardial infarction, heart problem, cerebrovascular disease, migraine, diabetes type I and II, and thyroid dysfunction), treatment for diabetes and hypothyroidism, and treatment with psychotropics (including lithium, valproate, lamotrigine, antidepressants, first- and second-generation antipsychotics, and central stimulants), lipid lowering agents, and antihypertensives. Some of the CMRIs had moderately to highly skewed distribution (with skewness more than + 0.5) according to Bulmer [36]. These variables were SBP at baseline, weight at follow-up, and TAG and hip circumference at baseline and follow-up. We log-transformed these skewed variables before imputation and back-transformed the imputed variables to the original scale [37]. We created 20 imputed datasets to reduce the variability of parameter estimates like mean, median, and standard deviation from the imputation process [37, 38].

We used linear mixed effects model with a random intercept to examine the differences in average annual change in CMRIs between patients and controls across the follow-up period. We adjusted the linear mixed effects model for sex, age at baseline, and for follow-up time because follow-up time varied both within and between the studied groups. We considered a two tailed P < 0.05 as statistically significant. We did not correct for multiple comparisons because of the positive intercorrelation between all the eight tested CMRIs. All analyses were conducted using SPSS Statistics (version 29).

## Results

At baseline, the cohort included 413 individuals with BDs and 56 controls. We excluded 6 individuals with BDs because of total missing of CMRIs´ data. The final baseline cohort thus comprised 407 persons with BDs and 56 controls. Of those, 63 persons with BDs and 42 controls participated at follow up.

Table 1 shows the characteristics of the study groups at baseline. The BDs group was younger and contained more women. In comparison with controls, the BDs group also had lower GAF-scores, fewer people who worked at least 50% of the time, fewer people with education levels more than 12 years, and a higher prevalence of somatic comorbidities. Although there were fewer smokers overall in the patient group, a higher proportion of them were moderate and heavy smokers than in the control group.

**Table 1:** Clinical characteristics of the study groups at baseline

	Patients	n	Controls	n
Women, n (%)	255 (63)	407	30 (54)	56
Age, mean ± SD, years	40 ± 13	407	43 ± 12	56
GAF symptom, mean ± SD	61 ± 10	402	89 ± 7	56
GAF function, mean ± SD	63 ± 10	402	89 ± 6	56
Smoking		366		56
Non-smoker, n (%)	317 (86.6)		47 (83.9)	
Light smoker (< 10 cigarettes per day), n (%)	18 (4.9)		7 (12.5)	
Moderate smoker (10–19 cigarettes per day), n (%)	17 (4.6)		2 (3.6)	
Heavy smoker (≥ 20 cigarettes per day), n (%)	14 (3.8)		0	
> 12 y of education, n (%)	183 (46)	397	32 (57)	56
Working more than 50%, n (%)	240 (60)	399	53 (96)	55
Somatic comorbidity				
Hypertension, n (%)	23 (5.7)	406	0	56
Angina pectoris, n (%)	0	406	0	56
Myocardial infarction, n (%)	0	406	0	56
Other heart problems, n (%)	0	406	0	56
Cerebrovascular disease, n (%)	1 (0.2)	406	0	56
Migraine, n (%)	13 (3.2)	406	1 (1.8)	56
Diabetes mellitus type II, n (%)	6 (1.5)	406	0	56
Diabetes mellitus type I, n (%)	3 (0.7)	406	0	56
Hypothyroidism, n (%)	69 (17.0)	406	0	56
Hyperthyroidism, n (%)	1 (0.2)	406	0	56
Disease duration, median (25–75 percentiles), years	20 (13–30)	406		
Age at first treatment with psychotropics, mean ± SD, years	28 ± 11	222		
Bipolar subtype, n (%)		407		
Bipolar I disorder	130 (32)			
Bipolar II disorder	226 (55)			
Bipolar disorder non-otherwise specified (NOS)	36 (9)			
Cyclothymia	15 (4)			
Prescribed psychotropics, n (%)		278		
Lithium	128 (46)			
Valproate	11 (4)			
Lamotrigine	123 (44)			
Antidepressants	155 (56)			
First and second-generation antipsychotics	97 (35)			
Central stimulants	7 (3)			

Abbreviations: GAF, global assessment of functioning; SD, standard deviation

### Baseline

Table 2 shows baseline CMRIs in the BDs group and controls. Individuals with BDs had significantly higher mean BMI (β = 0.14, p = 0.003) compared with the controls. The other CMRIs did not differ between the groups.

**Table 2:** Baseline comparisons of cardiometabolic risk indicators between patients and controls

CMRIs	Patients (n = 407)	Controls (n = 56)	T-test		Linear regression (adjusted for age and sex)	
			**Mean difference (95% CI)**	**P-value**	**Coefficient estimate**	**P-value**
WHR, mean ± SD	0.88 ± 0.08	0.89 ± 0.07	− 0.01 (− 0.03–0.01)	0.2	0.003	> 0.30
BMI, mean ± SD, kg/m^2^	26.9 ± 5.9	24.9 ± 3.6	2.1 (0.9–3.2)	< 0.001	0.14	0.003
SBP, mean ± SD, mm Hg	124.2 ± 14.4	126.2 ± 10.3	− 1.9 (− 5.1–1.1)	0.2	− 0.003	> 0.30
DBP, mean ± SD, mm Hg	76.8 ± 9.3	80.3 ± 8.7	− 3.4 (− 6.0 – − 0.9)	0.009	− 0.09	0.05
TAG, mean ± SD, mmol/L	1.1 ± 0.9	1.0 ± 0.5	0.1 (− 0.1–0.3)	0.2	0.06	0.2
TAG/HDL-C ratio, mean ± SD	0.9 ± 1.5	0.7 ± 0.6	0.2 (− 0.2–0.6)	> 0.30	0.06	0.2
TChol/HDL-C ratio, mean ± SD	3.6 ± 2.1	3.5 ± 1.5	0.2 (− 0.4–0.7)	> 0.30	0.04	> 0.30
Non-HDL-C, mean ± SD, mmol/L	3.5 ± 1.0	3.6 ± 1.0	− 0.1 (− 0.4–0.2)	> 0.30	− 0.005	> 0.30

Note

Comparisons are made using multiply imputed data.

Abbreviations: BMI, body mass index; CI, confidence interval; CMRIs, cardiometabolic risk indicators; DBP, diastolic blood pressure; HDL-C, plasma high-density lipoprotein-cholesterol; SBP, systolic blood pressure; SD, standard deviation; TAG, fasting plasma triacylglycerol; TChol, total plasma cholesterol; WHR, waist-to-hip ratio.

We conducted a sensitivity analysis with available cases (not using imputed data) to examine the robustness of the multiple imputation [39]. Estimates of mean values and standard deviations were preserved (Supplementary Table 1) except that patients had significantly higher TAG (β = 0.16, p = 0.002) and TAG/HDL-C ratio (β = 0.11, p = 0.04) than controls in the available cases analysis.

We examined if study persons who participated at follow-up differed at baseline from those that participated at baseline only. In patients, we found no statistically significant differences in sex and bipolar subtype between individuals who participated at follow-up and those who did not. Supplementary Tables 2 and 3 show the baseline mean values of CMRIs in study participants who did and did not participate at follow-up. In the BDs group, those who participated at follow-up had lower SBP (β = − 0.14, p = 0.003), but higher TAG (β = 0.16, p < 0.001) and non-HDL-C (β = 0.18, p < 0.001) than those who only participated at baseline (Supplementary Table 2). In the control group, there were no statistically significant differences in the baseline CMRIs between those that participated and not participated at follow-up (Supplementary Table 3).

### Time-group interaction

**Fig. 1:**
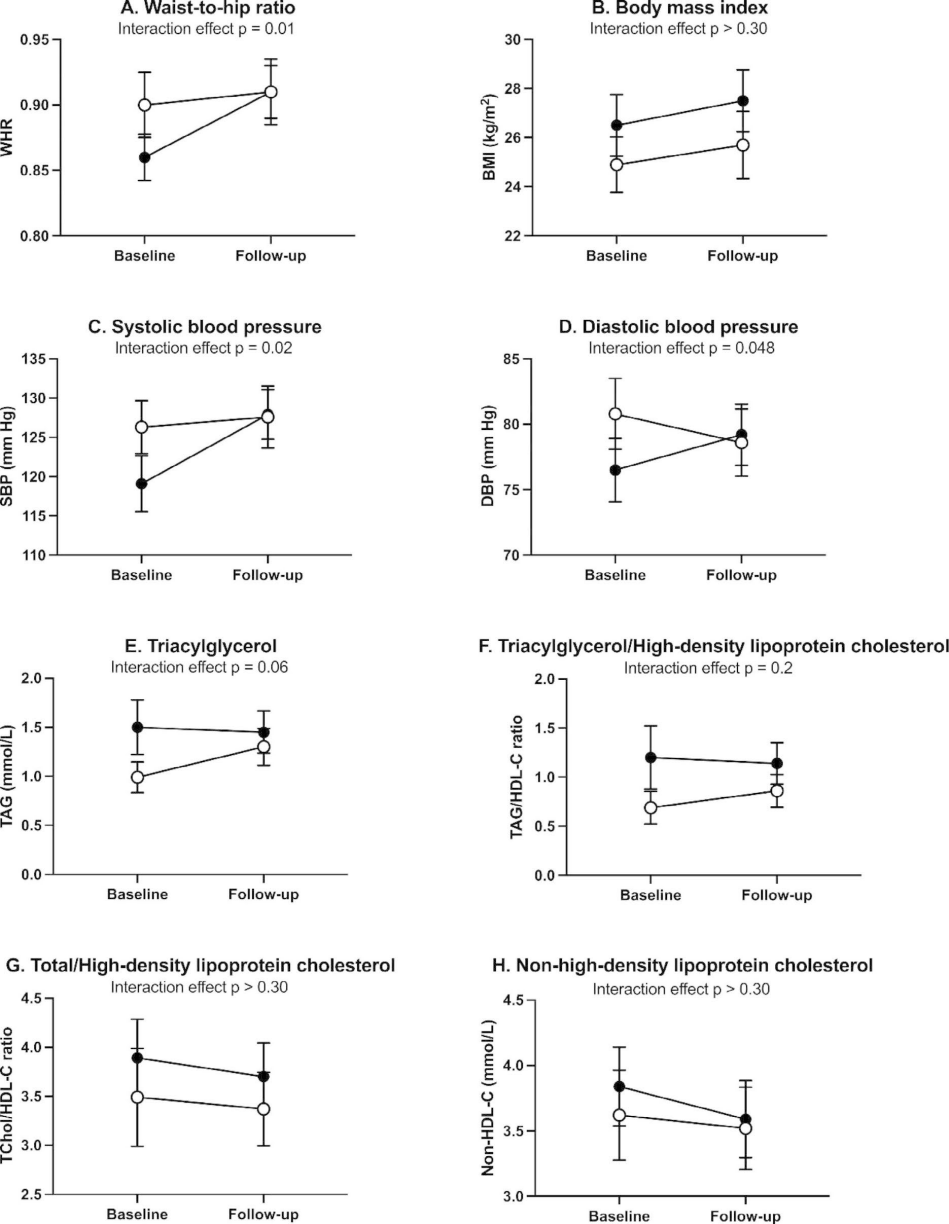
CMRI levels at baseline and follow-up in individuals with bipolar disorder (filled circles) and controls (open circles) Note Data are presented as means and 95% CI, comparisons are made using multiply imputed data. The significance values for the interaction effect in a linear mixed effects model adjusted for age at baseline, sex, and follow-up time **Abbreviations**: BMI, body mass index; DBP, diastolic blood pressure; SBP, systolic blood pressure; TAG, fasting plasma triacylglycerol; HDL-C, plasma high density lipoprotein cholesterol; TChol, total plasma cholesterol; WHR, waist-to-hip ratio

We tested group-by-time interaction-effects adjusted for follow-up time using linear mixed effects model and included only those who participated at both baseline and follow-up. We analyzed with and without adjusting for sex and age at baseline (Fig. 1A-H and Supplementary Table 4). Based on the difference in average annual change between the patient and the control group, we found an increase in patients relative to controls over time in WHR (0.004 unit/year, p = 0.01), DBP (0.6 mm Hg/year, p = 0.048), and SBP (0.8 mm Hg/year, p = 0.02). The time-group interaction was not statistically significant for the other CMRIs. We last performed a time-group interaction-effect analysis with available cases to examine the robustness of the multiple imputation (supplementary Table 5). The coefficient estimates were preserved in this sensitivity analysis, but the time-patient group interaction effect was no longer statistically significant for WHR (only 37 patients had available data on WHR at both baseline and follow-up).

## Discussion

We recently reported that individuals with BDs had higher mean baseline values for measures of total and central obesity, and a more atherogenic lipid profile than a control group. We also found a further worsening in central obesity and blood pressure in individuals with BDs relative to the controls over the follow-up period [28]. Here, we sought to replicate these findings in an independent case-control cohort with a similar study design. We first replicated a higher baseline level of BMI in the BDs group compared with the control group, but did not replicate statistically significant baseline differences in WHR, TAG, TAG/HDL ratio, TChol/HDL-C ratio, and non–HDL-C between the two groups. Second, we replicated the deterioration of WHR, SBP, and DBP in the patient group relative to the control group over the follow-up time.

In our previous study, we were cautious in interpreting the time-dependent increase in blood pressure in the patient group relative to the control group because the control group had higher baseline SBP and DBP than the patient group: We assumed a regression of blood pressure to the mean in the patient group. However, the findings regarding blood pressure were replicated in the present study, which suggests that the increase in systolic and diastolic blood pressure in the patient group reflects a true time-group interaction effect. This is also supported by increased WHR in the patient group because WHR has a strong direct relation to blood pressure [40].

In the analysis using imputed data at baseline, there were no statistically significant differences in the levels of TAG and TAG/HDL ratio between the patient group and controls. These results were at odds with what we expected and differed from the available case analysis. The available case analysis might, however, be falsely positive due to the large number of missing baseline data on TAG (113 patients) and TAG/HDL ratio (114 patients), which inflate the risk for a type I error [35]. Second, the low number of controls results in low statistical power, which also paradoxically increases the risk for false positive results [41]. The results of the imputed data are more robust because multiple imputation provides less biased effect size estimates and more accurate standard errors for hypothesis testing [34]. Moreover, we log-transformed the highly positively skewed TAG before imputation, which reduces the risk of false negative results [37]. Finally, we created 20 imputed datasets to reduce the variability of the parameter estimates resulting in smaller standard errors and narrower confidence intervals [37, 38].

Contrary to our previous results, we did not find any statistically significant baseline differences in CMRIs between the two groups except for BMI. This could be related to case-mix differences between the two cohorts. Another factor could be the lower prevalence of current cigarette smokers in the Gothenburg patient cohort since tobacco smoking has a dose response effect with direct relation with TAG and TChol and inverse relation with HDL-C [42]: In Stockholm, 31.7% of patients and 13.2% of controls were current smokers, whereas in Gothenburg, 13.4% of patients and 16.1% of controls were current smokers.

Like in our previous study, the observed group differences were small. However, we argue that the differences and changes in CMRIs between the two groups nevertheless are clinically important. The patient-control BMI difference at baseline was 2 kg/m^2^. The mean within-individual increases in the patient group were 0.05 units in WHR, 8.6 mm Hg in SBP, and 2.7 mm Hg in DBP. Even though these differences and changes were small and sub-threshold, small changes in CMRIs should become the focus of attention instead of the used clinical cut-offs [43]. First, because there is an increased risk of CVD even with changes as small as 0.01 unit of WHR [18] and one kg/m^2^ of BMI [19, 44, 45]. Further, there is a linear increase in CVD risk with higher SBP and DBP [46]. Second, the combination of small increases in several CMRIs leads to a synergistic effect [26, 27].

### Strengths and limitations

The strengths of this study include the longitudinal study design, the meticulous phenotyping, and the application of multiple imputation in dealing with missing values. There are also some limitations. First, we cannot exclude a potential selection bias in our study cohort because of lower prevalence of obesity (BMI ≥ 30 kg/m^2^) at baseline than would be expected in both the patient and control group. The prevalence of obesity in our patient group was 23%, which can be compared with the 33% prevalence in the Swedish National Quality Register for Bipolar Disorder in 2022 [47]. In our control group, the obesity prevalence was 9%, which can be compared with an estimated prevalence of 16% in the general population in 2020 [48]. Second, another source of selection bias is the lower SBP and higher TAG and non-HDL-C in the patient group that participated at baseline and follow-up compared with patients who participated at baseline only. Third, the low number of controls limits the statistical power [49]. Lastly, although study procedures were similar for both cohorts, we cannot exclude that results are influenced by time-dependent factors. Inclusion in the Gothenburg cohort began four years later than the Stockholm cohort and the inclusion period was longer. However, the inclusion of control groups in each cohort reduces the risk for bias.

## Conclusion

Both our current and previous study showed that central obesity, SBP, and DBP worsened over a relatively short time in individuals with BDs relative to controls. Thus, it is vital for clinicians to monitor and be aware of changes in CMRIs in persons with BDs and act proactively in preventing cardiometabolic diseases in this high-risk group.

## Electronic supplementary material

Supplementary tables

## Data Availability

According to the Swedish Public Access to Information and Secrecy Act, individual level data cannot be publicly available. Data used in this study are archived at University of Gothenburg.

## List of abbreviations

ADE: Affective disorder evaluation
AUDIT: Alcohol use disorders identification test
BDs: Bipolar disorders
BMI: Body mass index
CMRIs: Cardiometabolic risk indicators
CVD: Cardiovascular disease
DBP: Diastolic blood pressure
DSM-IV: Diagnostic and statistical manual of mental disorders- fourth edition
DUDIT: Drug use disorders identification test
GAF: Global assessment of functioning
HDL-C: High-density lipoprotein-cholesterol
LDL-C: Low-density lipoprotein-cholesterol
MAR: Missing at random
M.I.N.I.: Mini international neuropsychiatric interview
NOS: Bipolar disorder non-otherwise specified
SBP: Systolic blood pressure
SCID-I: Structured clinical interview for DSM-IV axis I disorders
STEP-BD: Systematic treatment enhancement program of bipolar disorder
TAG: Triacylglycerol
TChol: Total cholesterol
WHR: Waist-to-hip ratio
