# Supplementary material for: Cardiometabolic risk indicators in individuals with bipolar disorders: a replication study
Source: Diabetol Metab Syndr. 2023 Apr 3;15:69. doi: 10.1186/s13098-023-01044-7 (PMC10069119; doi:10.1186/s13098-023-01044-7)
Supplement: Supplementary file 1 — Supplementary tables [file 13098_2023_1044_MOESM1_ESM.doc]

**Appendix**

**Supplementary table 1. Baseline comparison of cardiometabolic risk indicators between patients and controls**

| **CMRIs** | **Patients** | **n** | **Controls** | **n** | **T-test** | | **Linear regression**  **(adjusted for age and sex)** | |
| --- | --- | --- | --- | --- | --- | --- | --- | --- |
|  |  |  |  |  | **Mean difference (95% CI)** | **P-value** | **Coefficient estimate** | **P-value** |
| **WHR, mean ± SD** | 0.88 ± 0.08 | 229 | 0.89 ± 0.07 | 56 | - 0.01 (- 0.03 – 0.01) | > 0.30 | 0.04 | > 0.30 |
| **BMI, mean ± SD, kg/m^2^** | 26.9 ± 5.9 | 401 | 24.9 ± 3.6 | 56 | 2.1 (0.9 – 3.2) | < 0.001 | 0.14 | 0.003 |
| **SBP, mean ± SD, mm Hg** | 123.6 ± 14.8 | 338 | 126.2 ± 10.3 | 56 | - 2.6 (- 5.7 – 0.6) | 0.1 | - 0.02 | > 0.30 |
| **DBP, mean ± SD, mm Hg** | 77.5 ± 9.6 | 338 | 80.3 ± 8.7 | 56 | - 2.8 (- 5.4 – - 0.07) | 0.04 | - 0.07 | 0.2 |
| **TAG, mean ± SD, mmol/L** | 1.4 ± 0.9 | 294 | 1.0 ± 0.5 | 55 | 0.3 (0.2 – 0.5) | < 0.001 | 0.16 | 0.002 |
| **TAG/HDL-C ratio, mean ± SD** | 1.2 ± 1.8 | 293 | 0.7 ± 0.6 | 55 | 0.4 (- 0.07 – 0.9) | 0.09 | 0.11 | 0.04 |
| **TChol/HDL-C ratio, mean ± SD** | 3.7 ± 2.4 | 294 | 3.5 ± 1.6 | 55 | 0.3 (- 0.4 – 0.9) | > 0.30 | 0.07 | 0.2 |
| **Non-HDL-C, mean ± SD, mmol/L** | 3.6 ± 1.1 | 294 | 3.6 ± 1.1 | 55 | - 0.02 (- 0.3 – 0.3) | > 0.30 | 0.05 | 0.3 |
| **Note**  **Comparisons are made using available case analysis.**  **Abbreviations: BMI, body mass index; CI, confidence interval; CMRIs, cardiometabolic risk indicators; DBP, diastolic blood pressure; HDL-C, plasma high-density lipoprotein-cholesterol; SBP, systolic blood pressure; SD, standard deviation; TAG, fasting plasma triacylglycerol; TChol, total plasma cholesterol; WHR, waist-to-hip ratio.** | | | | | | | | |

**Supplementary table 2. Baseline comparison of cardiometabolic risk indicators between patients who participated in follow-up with patients who didn´t**

| **CMRIs** | **Participated at baseline and follow-up (n=63)** | **Participated at baseline only (n=344)** | **T-test** | | **Linear regression**  **(adjusted for age and sex)** | |
| --- | --- | --- | --- | --- | --- | --- |
|  |  |  | **Mean difference (95% CI)** | **P-value** | **Coefficient estimate** | **P-value** |
| **WHR, mean ± SD** | 0.86 ± 0.07 | 0.88 ± 0.08 | - 0.02 (- 0.04 – 0.001) | 0.06 | - 0.06 | 0.09 |
| **BMI, mean ± SD, kg/m^2^** | 26.5 ± 5.0 | 27.0 ± 6.1 | - 0.5 (- 2.1 – 1.1) | > 0.30 | - 0.03 | > 0.30 |
| **SBP, mean ± SD, mm Hg** | 119.1 ± 14.2 | 125.1 ± 14.2 | - 5.9 (- 9.8 – - 2.2) | 0.002 | - 0.14 | 0.003 |
| **DBP, mean ± SD, mm Hg** | 76.5 ± 9.6 | 76.9 ± 9.2 | - 0.4 (- 2.9 – 2.1) | > 0.30 | - 0.004 | > 0.30 |
| **TAG, mean ± SD, mmol/L** | 1.5 ± 1.1 | 1.1 ± 0.9 | 0.4 (0.1 – 0.7) | 0.01 | 0.16 | < 0.001 |
| **TAG/HDL-C ratio, mean ± SD** | 1.2 ± 1.3 | 0.9 ± 1.6 | 0.3 (- 0.1 – 0.7) | 0.2 | 0.08 | 0.09 |
| **TChol/HDL-C ratio, mean ± SD** | 3.9 ± 1.6 | 3.6 ± 2.2 | 0.3 (- 0.2 – 0.9) | 0.3 | 0.07 | 0.1 |
| **Non-HDL-C, mean ± SD, mmol/L** | 3.8 ± 1.2 | 3.4 ± 0.9 | 0.2 (0.2 – 0.8) | 0.005 | 0.18 | < 0.001 |
| **Note**  **Comparisons are made using multiply imputed data.**  **Abbreviations: BMI, body mass index; CI, confidence interval; CMRIs, cardiometabolic risk indicators; DBP, diastolic blood pressure; HDL-C, plasma high-density lipoprotein-cholesterol; SBP, systolic blood pressure; SD, standard deviation; TAG, fasting plasma triacylglycerol; TChol, total plasma cholesterol; WHR, waist-to-hip ratio.** | | | | | | |

**Supplementary table 3. Baseline comparison of cardiometabolic risk indicators between controls who participated in follow-up with controls who didn´t**

| **CMRIs** | **Participated at baseline and follow-up (n=42)** | **Participated at baseline only (n=14)** | **T-test** | | **Linear regression**  **(adjusted for age and sex)** | |
| --- | --- | --- | --- | --- | --- | --- |
|  |  |  | **Mean difference (95% CI)** | **P-value** | **Coefficient estimate** | **P-value** |
| **WHR, mean ± SD** | 0.89 ± 0.08 | 0.88 ± 0.04 | 0.02 (- 0.02 – 0.06) | 0.3 | 0.11 | 0.2 |
| **BMI, mean ± SD, kg/m^2^** | 24.9 ± 3.6 | 24.6 ± 3.8 | 0.4 (- 1.9 – 2.6) | > 0.30 | 0.03 | > 0.30 |
| **SBP, mean ± SD, mm Hg** | 126.3 ± 10.8 | 125.7 ± 9.2 | 0.6 (- 5.8 – 7.0) | > 0.30 | - 0.03 | > 0.30 |
| **DBP, mean ± SD, mm Hg** | 80.8 ± 8.7 | 78.6 ± 8.9 | 2.3 (- 3.1 – 7.7) | > 0.30 | 0.07 | > 0.30 |
| **TAG, mean ± SD, mmol/L** | 0.9 ± 0.5 | 1.2 ± 0.6 | - 0.2 (- 0.5 – 0.1) | 0.3 | - 0.14 | 0.3 |
| **TAG/HDL-C ratio, mean ± SD** | 0.7 ± 0.5 | 0.9 ± 0.9 | - 0.2 (- 0.6 – 0.2) | > 0.30 | - 0.12 | > 0.30 |
| **TChol/HDL-C ratio, mean ± SD** | 3.5 ± 1.6 | 3.4 ± 1.4 | 0.1 (- 0.8 – 1.1) | > 0.30 | 0.03 | > 0.30 |
| **Non-HDL-C, mean ± SD, mmol/L** | 3.6 ± 1.1 | 3.5 ± 0.9 | 0.1 (- 0.5 – 0.8) | > 0.30 | 0.01 | > 0.30 |
| **Note**  **Comparisons are made using multiply imputed data.**  **Abbreviations: BMI, body mass index; CI, confidence interval; CMRIs, cardiometabolic risk indicators; DBP, diastolic blood pressure; HDL-C, plasma high-density lipoprotein-cholesterol; SBP, systolic blood pressure; SD, standard deviation; TAG, fasting plasma triacylglycerol; TChol, total plasma cholesterol; WHR, waist-to-hip ratio.** | | | | | | |

**Supplementary table 4. Interaction between the follow-up time and the patient/control-variable with and without adjusting for confounders**

| **CMRIs** | **Participants** | **Coefficient estimate^*^** | **95% CI** | **P-value^†^** | **Coefficient estimate (adjusted)** | **95% CI (adjusted)** | **P-value^‡^** |
| --- | --- | --- | --- | --- | --- | --- | --- |
| **WHR** | Controls | 0.001 | - 0.002 – 0.004 | > 0.30 | 0.001 | - 0.002 – 0.004 | > 0.30 |
|  | Patients | 0.004 | 0.001 – 0.008 | 0.01 | 0.004 | 0.001 – 0.008 | 0.01 |
| **BMI** | Controls | 0.1 | - 0.005 – 0.2 | 0.06 | 0.1 | - 0.005 – 0.2 | 0.06 |
|  | Patients | 0.005 | - 0.1 – 0.2 | > 0.30 | 0.005 | - 0.1 – 0.2 | > 0.30 |
| **SBP** | Controls | 0.2 | - 0.4 – 0.7 | > 0.30 | 0.2 | -0.4 – 0.8 | > 0.30 |
|  | Patients | 0.9 | 0.2 – 1.6 | 0.02 | 0.8 | 0.1 – 1.5 | 0.02 |
| **DBP** | Controls | - 0.3 | - 0.8 – 0.2 | 0.2 | - 0.3 | - 0.8 – 0.2 | 0.3 |
|  | Patients | 0.6 | 0.02 – 1.2 | 0.04 | 0.6 | 0.006 – 1.2 | 0.048 |
| **TAG** | Controls | 0.04 | 0.003 – 0.08 | 0.04 | 0.04 | 0.003 – 0.08 | 0.04 |
|  | Patients | - 0.04 | - 0.09 – 0.001 | 0.06 | - 0.04 | - 0.09 – 0.001 | 0.06 |
| **TAG/HDL-C ratio** | Controls | 0.02 | - 0.01 – 0.06 | 0.2 | 0.02 | - 0.01 – 0.06 | 0.2 |
|  | Patients | - 0.03 | - 0.08 – 0.01 | 0.2 | - 0.03 | - 0.08 – 0.01 | 0.2 |
| **TChol/HDL-C ratio** | Controls | - 0.02 | - 0.06 – 0.03 | > 0.30 | - 0.02 | - 0.06 – 0.03 | > 0.30 |
|  | Patients | - 0.01 | - 0.07 – 0.05 | > 0.30 | - 0.01 | - 0.07 – 0.05 | > 0.30 |
| **Non-HDL-C** | Controls | - 0.01 | - 0.05 – 0.03 | > 0.30 | - 0.01 | - 0.05 – 0.03 | > 0.30 |
|  | Patients | - 0.02 | - 0.07 – 0.03 | > 0.30 | - 0.02 | - 0.07 – 0.03 | > 0.30 |
| ***** Controls´ rows show average annual change in estimates during the follow-up period for the control group. Patients´ rows show estimates for the difference in average annual change between the patient group and the control group.  **†** P-value adjusted for follow-up time.  **‡** P-value adjusted for age at baseline, sex, and follow-up time.  **Note**  Comparisons are made using multiply imputed data.  **Abbreviations:** BMI, body mass index; CI, confidence interval; CMRIs, cardiometabolic risk indicators; DBP, diastolic blood pressure; HDL-C, plasma high density lipoprotein cholesterol; SBP, systolic blood pressure; SD, standard deviation; TAG, fasting plasma triacylglycerol; TChol, total plasma cholesterol; WHR, waist-to-hip ratio. | | | | | | | |

**Supplementary table 5. Interaction between the follow-up time and the patient/control-variable with and without adjusting for confounders**

| **CMRIs** | **Participants**  **(no. of observations)** | **Coefficient estimate^*^** | **95% CI** | **P-value^†^** | **Coefficient estimate (adjusted)** | **95% CI (adjusted)** | **P-value^‡^** |
| --- | --- | --- | --- | --- | --- | --- | --- |
| **WHR** | Controls (40) | 0.002 | - 0.001 – 0.004 | 0.3 | 0.002 | - 0.001 – 0.004 | 0.3 |
|  | Patients (37) | 0.003 | - 0.001 – 0.007 | 0.09 | 0.003 | - 0.001 – 0.007 | 0.1 |
| **BMI** | Controls (40) | 0.08 | - 0.03 – 0.2 | 0.2 | 0.07 | - 0.03 – 0.2 | 0.2 |
|  | Patients (62) | 0.05 | - 0.08 – 0.2 | > 0.30 | 0.05 | - 0.08 – 0.2 | > 0.30 |
| **SBP** | Controls (41) | 0.2 | - 0.4 – 0.7 | > 0.30 | 0.2 | - 0.4 – 0.7 | > 0.30 |
|  | Patients (58) | 0.9 | 0.2 – 1.7 | 0.01 | 0.9 | 0.2 – 1.7 | 0.01 |
| **DBP** | Controls (41) | - 0.3 | - 0.8 – 0.1 | 0.2 | - 0.3 | - 0.8 – 0.1 | 0.2 |
|  | Patients (58) | 0.6 | 0.03 – 1.2 | 0.04 | 0.6 | 0.03 – 1.2 | 0.04 |
| **TAG** | Controls (37) | 0.02 | - 0.02 – 0.06 | 0.2 | 0.02 | - 0.01 – 0.06 | 0.2 |
|  | Patients (63) | - 0.03 | - 0.07 – 0.02 | 0.2 | - 0.03 | - 0.07 – 0.02 | 0.2 |
| **TAG/HDL-C ratio** | Controls (35) | 0.02 | - 0.03 – 0.06 | > 0.30 | 0.02 | - 0.02 – 0.06 | > 0.30 |
|  | Patients (63) | - 0.03 | - 0.07 – 0.02 | > 0.30 | - 0.03 | - 0.07 – 0.02 | 0.3 |
| **TChol/HDL-C ratio** | Controls (35) | - 0.002 | - 0.05 – 0.05 | > 0.30 | - 0.0003 | - 0.05 – 0.05 | > 0.30 |
|  | Patients (63) | - 0.02 | - 0.08 – 0.04 | > 0.30 | - 0.03 | - 0.08 – 0.03 | > 0.30 |
| **Non-HDL-C** | Controls (35) | 0.004 | - 0.04 – 0.05 | > 0.30 | 0.004 | - 0.04 – 0.05 | > 0.30 |
|  | Patients (63) | - 0.04 | - 0.08 – 0.02 | 0.2 | - 0.03 | - 0.08 – 0.01 | 0.2 |
| ***** Controls´ rows show average annual change in estimates during the follow-up period for the control group. Patients´ rows show estimates for the difference in average annual change between the patient group and the control group.  **†** P-value adjusted for follow-up time.  **‡** P-value adjusted for age at baseline, sex, and follow-up time.  **Note**  Comparisons are made using available case analysis.  **Abbreviations:** BMI, body mass index; CI, confidence interval; CMRIs, cardiometabolic risk indicators; DBP, diastolic blood pressure; HDL-C, plasma high density lipoprotein cholesterol; SBP, systolic blood pressure; SD, standard deviation; TAG, fasting plasma triacylglycerol; TChol, total plasma cholesterol; WHR, waist-to-hip ratio. | | | | | | | |
